# Hospital and Institutional News

**Published:** 1917-06-02

**Authors:** 


### HOSPITAL AND INSTITUTIONAL NEWS.

### AMERICAN MEDICAL UNITS FOR THE FRONT.

EVEN through the most trying times it is certain that the great bulk of the people in this country have had no real doubt as to the issue of the war. Timorous voices have been heard at times, but they have been both few and feeble. Nor have they, as time has passed, made any appreciable growth either in number or in strength. Indeed, one of the most remarkable features of this time of tense national history has been the people's quiet, resolute, undemonstrative confidence alike in the righteousness of their cause and the certainty of victory. On the entrance of America as an active participator in the great struggle, this confidence has become still more secure. Especially is it felt that what America sets out to do will be done thoroughly and with a will to victory that nothing can daunt. There will be delays, as we know from our own experience, but our people have the secure conviction that on the other side of the Atlantic there is a swift resolve to organise victory, and a stern determination to achieve it. In the nature of things many arrangements resulting from the co-operation of America cannot be made public, but the interest in what has been told is intense. This is conspicuously true of the intimation that medical units, including surgeons and nurses, have reached this country on their way to the Front, and never did His Majesty the King express more fully the mind of the nation than when he, together with the Queen, gave a personal reception and welcome to "the first detachment of the American Army which has landed on our shores since your great Republic resolved to join in the world-struggle for the ideals of civilisation." It has not been proclaimed loudly, but many people know how sustained, effective, and large-hearted has been the surgical and nursing work contributed by Americans to the sick and wounded in France. Now they come with a new status, and we rejoice the more to have them at our side. If we do not display a very full emotion, our Transatlantic friends must take our calm as an index of our conviction that the new arrangement is a strictly natural one. It seems to us in the very nature of things that America should be urgent for a cause which, as we see it, is the cause of righteousness and of liberty, and we accept as fit and proper and inevitable an event which shows our anticipation to be well justified. Particularly in these columns may be expressed a word of welcome to doctors and nurses who come with the tradition of American efficiency and chivalry and helpfulness. The help and co-operation which they bring in the midst of intense national stress and individual suffering beyond the power of words to express have a special appeal, for the voice has none of the tones of the stranger and is familiar to us as those of our own household.

### AN INSTITUTIONAL LAW-SUIT.

THE Lancashire Chancery Court has been called upon to determine an interesting point regarding a bequest to the Oldham Royal Infirmary. The matter arose in connection with the estate of the late Mr. Henry L. Hargreaves, of Oldham, who died in August 1914. He left considerable estate, the total personalty amounted to over £15,000, and, after making other provisions, he devised all his residuary real and personal estate upon trust to his executors and trustees for the purpose of building a convalescent home in connection with the Oldham Royal Infirmary. The surviving trustee presented a petition, raising the question whether the disposition of the estate constituted a valid charitable gift, and whether it was void as transgressing the rules with regard to perpetuities. Mr. Talbot, for the petitioner, explained that there was a proviso in the will that the money was not to be handed over until a scheme was formulated, and that in the meantime it should accumulate. A scheme had been formulated since the presentation of the petition, to which the petitioner had given his assent. For the heir-at-law and next-of-kin Mr. Roby argued that the gift to the charity was void, but Mr. Grant, K.C., for the infirmary, submitted that the gift, notwithstanding the proviso in the will, was absolute to the infirmary, and that the rule as to perpetuity had no effect, the dedication being complete. This view was supported also by the Attorney-General for the County Palatine (Mr. R. A. McCall, K.C.). Vice-Chancellor Stewart-Smith, K.C., held that the bequest to the Oldham Infirmary was a charitable gift, and gave judgment accordingly.

### THE ANGLO-RUSSIAN HOSPITAL AT PETROGRAD

MR. STEPHEN PAGET is already famous as an essayist of no ordinary calibre, and his delightful contribution to hospital lore in "A Short Account of the Anglo-Russian Hospital, Petrograd," is written in his most attractive style. This book, on sale at the Russian Exhibition and through the usual channels, possesses the excellent quality of avoiding too much in the way of geographical and statistical information. The shillings that it will readily draw from an appreciative public will substantially augment the funds of the Anglo-Russian Hospital, to which they are to be devoted. Mr. Paget describes the Russian soldier as a big child, and tells how he saw one hiding under his bed with the object of bewildering the ward sister. "Never," says the author, "till I came to Russia had I seen what we call a religious country"; the patients pray long at their bedside, using many acts of observance and reverence. Apropos of prayer, a story may be taken from the book of one of the sisters who, after a particularly uneventful and peaceful day, remarked: "And to think that all this time I'm being prayed for in two villages at home as one in special danger!" Mr. Paget claims that the hospital has done more than care for the wounded—it has demonstrated the good will of our country towards Russia.

### THE EARL OF LICHFIELD'S OPPORTUNITY.

ARE hospital managers as much alive as they ought to be to the duty of publicly recording the names of those of their staffs who have fallen in the war? Tradition and its atmosphere is the breath of life to institutions, and managers should remember that nothing attracts loyalty better than those witnesses to past service which prompt its continuance and make the new generations realise that they have a heritage to hand on to their successors. Not long ago, for example, the Staffordshire General Infirmary lost two former house surgeons, Dr. Peel and Dr. Wyllie, both of whom have died from wounds received on active service. So far no hint has reached us that the hospital proposes to honour their memory and perpetuate their example. But the connection of these men with their hospital and the occasion of their deaths are too precious to be allowed to pass away. We hope that the Earl of Lichfield and Mr. R. Battle, the hospital's secretary, will give the matter their attention. It is their privilege to see that the walls where Dr. Peel and Dr. Wyllie worked remember them.

### THE MOST CHRISTIAN OF INSTITUTIONS.

THE Dean of Norwich lately preached in the cathedral at a service of thanksgiving for the work of the Red Cross. In the course of his address he described the care of the sick as one of the chief fruits of the Christian spirit, and went on to commend hospitals as the most typically Christian of all our benevolent institutions. His view once again emphasises the close tie which has always existed between the voluntary hospitals and the Churches, from the day when the religious Orders provided the chief institutions for healing the sick to modern times, when the late Canon Miller of Birmingham instituted the first Hospital Sunday. The tie is important because one of its results is the placing of the patient's good in the foreground, and relegating science to its proper place as a means and not an end which in itself justifies every experiment. The clergy, like most people, are apt to take this for granted, but they may take pride in the fact that institutions, like hospitals, can only be kept in the path of virtue so long as the compassion which prompted their creation remains unalloyed by professionalism. Without a sense of honour or religion, the pursuit of science degenerates, as our pre-war articles on German hospitals showed, into an engine of cruelty. That we have largely escaped this is due to the influence of the Christian spirit working through the voluntary system.

### A NOTTINGHAMSHIRE "STAR AND GARTER."

So as to carry into practical shape a suggestion by the Duchess of Portland for the establishment of a home for Nottinghamshire sailors and soldiers who may be permanently paralysed in the war, the Duke of Portland last week purchased for £2,100 a commodious residence, Ellerslie House, on Gregory Boulevard, Nottingham. The house overlooks Sherwood Forest, has beautiful gardens and a flat roof, and when lifts have been installed and various minor alterations carried out, it will prove ideal for its purpose. Accommodation will be provided for at least forty men, and the institution will—in so far as Nottinghamshire patients are concerned—relieve pressure at the Star and Garter Hospital, Richmond. The idea of the Duchess of Portland is that the example of Nottingham may be followed generally, and that ultimately a similar home shall be established in every county. A public appeal for support may be issued later, and, following an interview which Mr. Frederick Acton, on behalf of the Duchess, had with the Pensions Minister, it is hoped that Government help will be forthcoming towards the cost of maintenance.

### THE PRINCE OF WALES' HOSPITAL FOR LIMBLESS SAILORS AND SOLDIERS, CARDIFF.

A MEETING of the council of the Prince of Wales' Hospital, Cardiff, was held at the hospital on Thursday, May 24, under the presidency of Mrs. Lloyd George. In addition to Cardiff members of the council, there were also present Mrs. Gwynne Holford and Surgeon-General M. W. Russell, Deputy Director-General of the Army Medical Service. Prior to the council meeting the party inspected the hospital and were specially interested in the limb workshops and the parade hall. In the latter were the first ten men to be fitted with limbs made at the hospital. The Commandant (Miss Deacon) and Lieut.-Col. Lynn Thomas were congratulated by Mrs. Gwynne Holford and Surgeon-General Russell upon the planning and general arrangements of the hospital and upon the thorough way in which it was being equipped. The generosity of Mr. W. Percy Miles and Mr. J. P. Cadogan has enabled the scheme to be undertaken in a comprehensive manner. Preliminary arrangements have been made by the council for the formal opening of the hospital later in the summer, when the staff house and the officers' quarters are completed.

### HOSPITAL COLLECTOR KILLED BY THE GERMANS.

ONE of the victims of the severe aeroplane attack on a South-East town last week was a little girl who, by her own efforts, had collected over £63 for the local hospital. The solemn humbug of censorship, as applied to a fact equally well-known to the Germans and most English folk, does not permit us to record the name of this inoffensive victim of enemy brutality. Her portrait appears in one of the popular picture-papers—a photograph taken evidently just after she had made one of her successful efforts to direct practical public sympathy to the hospital of the town. She appears with a pile of six collecting-boxes in front of her, and, the terrible fact may or may not have been noticed by the censor, each box has on it the name of the hospital but not that of the town which the hospital serves. Now, even if we did not know where the raid took place, the English official account and the German wireless statement give a choice between two towns. The title of the hospital should make our information complete; but on looking into the matter a little more closely it is found that there is a hospital of exactly the same name in each of two large towns in the South-East of England. So, apparently, the censor is not so unobservant after all.

### R.A.M.C. NOMENCLATURE.

THE title of a correspondent to the *Midland Medical Journal*—"The M.O. to a V.A.D."—is in keeping with the subject of his contribution, the descriptions of military diagnoses, for purposes of classification, as represented by abbreviations. In alluding to the curious misapprehensions arising, he tells of a man coming to a medical meeting and telling his professional brethren that he had a case of "D.A.H." under his care, but that he could not find out what on earth was the matter with the patient. A witty Irishman present quickly attempted to solve the difficulty by remarking, "Shure! That's easy. D.A.H., d——d awful hæmorrhoids." It was not for some days after that the doctor in charge of the case found the clue to the mystery in disordered action of the heart. V.D.H. (valvular disease of the heart) is criticised because there is no indication as to which valve is involved. P.O.U.O. (pyrexia of unknown origin) is considered a little too indefinite. I.C.T., meaning inflammation of the connective tissue, is suggested as being a diagnosis that at times is hopelessly inadequate. But the writer of the paper, in poking a little harmless fun at the R.A.M.C., and suggesting that the sublime is ever near to the ridiculous, does not conceal his admiration for the magnificent work which the Corps has accomplished.

### EAST END SPORTSMEN.

THANKS to Mr. H. Gosling, L.C.C., and to Mr. A. Ansell, the sportsmen of the East End have come to the rescue of the East London Hospital for Children, Shadwell, and the proceeds of their games seem likely to be an annual source of income to it. As a result of a football match between teams representing the Thames Watermen and Lightermen and the Royal Engineers, Inland Water Transport Section, a sum of £100 has been handed to Mr. H. Machin, the hospital's chairman. Lately, in fact, a double presentation took place. Mr. Machin received the cheque, and the winning team, that of the watermen, the silver cup presented by Mr. Gosling, together with a gold-centre medal for each member. The decision to make an athletic contest an annual one led Mr. Machin to point out that if not less than £50 a year were collected in this fashion a cot could be named after the event or after the founders. By this means we hope that Mr. F. Stanley Cheer, the hospital's secretary, may receive an annual sum for his hospital, and that the sporting instincts of the watermen and their fellow-workers may be encouraged.

### A NEW HOSPITAL FOR BRISTOL.

MR. W. MELVILLE WILLS, president of the Bristol Homœopathic Hospital, has offered to present a new building, at a cost of £20,000, in memory of his son, the late Captain Bruce Melville Wills. It is estimated that a similar sum will be required to purchase a site and equip the new building. The site will probably be in the neighbourhood of Redland Green, and the architects, Messrs. Oatley and Lawrence, have drawn their plans for a hospital of forty beds. It is hoped to include some private wards, with special provision for those too poor to pay the fees of a nursing home and not poor enough for free treatment. The first step, however, is to raise the £20,000 required by the board for the purchase of the site and the provision of the equipment. The board has not hesitated to appeal for this sum, and Bristol is easily capable of providing it.

### A VALUABLE EXTENSION.

MR. STANLEY GIBBS, who was a member of the Stock Exchange before he joined the Naval Air Service, has lent his house adjoining the Brondesbury Park Military Hospital to that institution as an annexe for the duration of the war. Its grounds of four acres will be as much appreciated by the patients as the house itself will be by the authorities, for it enables another fifty patients to be accommodated. Mr. Alfred Butt, who opened the new wing as it is called, was publicly thanked by Colonel D'Arcy Power and Colonel Matthews for the result of the concert lately held on the hospital's behalf at the Palace Theatre, at which a sum of £930 was raised.

### THE ABSENT AMBULANCE.

NOT for the first time the London County Council ambulance service has been lately criticised for alleged negligence. The first cause in the recent complaint concerned the refusal of those in charge of an ambulance to remove from King's College Hospital (to which they had brought another case) a woman seriously injured in an accident, for whom there was no available room. On being requested to take her to Newington Infirmary those in charge of the ambulance declared that the regulations forbade them to do so. Mr. W. H. Ecroyd, at a recent Council meeting, referred to the case of Mr. H. J. Glanville, M.P., who, he declared, had to wait for an hour in the roadway, when suffering from a fractured thigh, before the ambulance arrived. Mr. Dove excused the ambulance on the ground that delays were sometimes inevitable owing to the ambulances being out when called for, or to temporary hitches with the telephone, but Mr. Ecroyd affirmed that he could quote several instances which would prove the necessity for an immediate overhauling of the service. Meantime Mr. Dove has issued fresh instructions which he hopes will prevent further complaints.

### A NEW FORM OF ECONOMY.

MORE than one blow has been struck at the survival of petty patronage, which chiefly subsists in the evil practice of subscribers' votes, and the less evil, but not satisfactory, use of recommendations. The latter are now often returned to the secretary's office of the hospital which issues them, but a further step towards their abolition has been taken at the Essex County Hospital, where, from motives of economy, the committee has found it necessary to discontinue forwarding them to subscribers, unless the latter particularly request that they should be sent. The Rev. E. Spurrell, the hospital's chairman, has stated that some economy has been brought about in this way, which is to be welcomed rather for its effect on the issue of these letters than for any saving in which it may result. It is well to note that he does not complain that subscribers have fallen off through the discontinuance of the old system, and this fact once again encourages the hope that even the odious and wasteful system by which subscribers have the right to vote at elections to a charity will one day be discontinued as a stupid and unnecessary bait for subscriptions which do not really depend upon it.

### CASH OR CREDIT?

It certainly adds to the book-keeping of a hospital and necessitates a much larger petty-cash "float" than in times of peace, but the system of cash for certain goods has its advantages. The housekeeper or steward now sees some of the stores before buying and does not so extensively rely on orders sent by post or telephone. In the circumstances it is much easier to reject supplies not up to the mark, and where the cash system prevails, deliveries which are "just good enough" are not so often accepted. There is one tradesman who buys vegetables (potatoes surprisingly often) and fruit in the market, and takes them round to the institutions in the West Central district, accepting a reasonable profit on his outlay. The housekeeper may be seen, occasionally, her cap and apron agitated by the breeze, peeping into the sacks on the tail-board of a waggon or examining a sample basket of apples or onions. Then, again, comes the all-too-frequent occasion when the margarine or bacon delivery has been deficient, and someone in the commissariat department has to visit the neighbouring shops, buying for cash across the counter. We believe that it is the custom, not only in war-time, of some of the clubs to send a buyer in the early morning to Billingsgate, Smithfield, and Covent Garden to obtain the day's requirements, and the goods there bought for cash are immediately taken away. Quality is assured, and we understand that the system from the economy point of view has distinct points. It would be interesting to learn if this method of buying is ever practised by the hospitals of our larger cities.

### COTTAGE HOSPITALS AND THE WAR.

Cottage hospitals have often a roll of honour which is remarkable for institutions of their size, and the fact is worth mentioning in days when the big institutions and the new military hospitals almost monopolise public attention. The Willesden Cottage Hospital, for example, possesses a treasurer, Mr. C. S. Gibbs, who has now received his commission as Lieutenant-Commander in the Navy, and the board, rather than lose touch with him, has declined his resignation, but has accepted the offer of one who formerly assisted him to act until Mr. Gibbs returns to civil life again. The hospital has managed to do plenty of work for soldiers, and has received a request to increase the number of beds placed at their disposal.

### THE ROLL OF HONOUR HOSPITAL.

A very influentially signed appeal for the rebuilding and endowment scheme of the Women's Hospital for Children, Harrow Road, which is changing its name to the Roll of Honour Hospital, has been issued through the public press. Sir John Jellicoe, Sir William Robertson, Sir Alfred Keogh, Lord Knutsford, and others have signed the appeal, which aims at securing donations of one thousand pounds to endow each of the fifty cots in the proposed new hospital. "For the last five years," says the letter, the hospital "has been carried on in temporary accommodation which is quite inadequate and unsuitable," a verdict which The Hospital can heartily endorse (see the report on the Women's Hospital for Children, The Hospital, January 31, 1914, p. 481). Besides the fifty gifts of a thousand pounds each which it is hoped to obtain, smaller donations are also asked to complete the total of £80,000 to £100,000 which the new hospital is expected to require for building and endowment.

### THE TREATMENT OF NEURASTHENICS.

A scheme is now in course of preparation by the War Office authorities to provide for the accommodation in Territorial hospitals of a certain number of men suffering from neurasthenia, shell shock, and allied war neuroses. The system, if proved to be practicable, will be a considerable advance on the present methods of dealing with this class of case. Briefly, the men, after having been examined by a board of specialists, will be classified according to their wage-earning capacity per hour. The maximum, *i.e.*, that of a normal person, being considered as 6d., the men will be graded in sets rising by 1½d., so that those of approximately the same capacity will be together. The work is to consist of light and, as far as possible, attractive agricultural labour, more especially the tending of gardens, etc., under the supervision of expert horticulturists. There is little doubt that many men would benefit incalculably by having their thoughts diverted from their own condition by healthful employment under the ideal hygienic conditions of open air and rural surroundings.

### AN EXCELLENT STORY.

If the following story were not vouched for as authentic by the *Guy's Hospital Gazette*, it would sound too good to be true. Two servant-girls, lately arrived from the country, finding themselves in consultant land, rang the bell of a well-known London anæsthetist and insisted on seeing him. On entering the consulting-room one of the young women said, "Please, sir, I want you to pull out my tooth." The doctor was very amused, and informed the girl that he did not draw teeth, but, seeing her in evident pain and noticing her swollen face, he decided to do the kind thing for her. He therefore rang up a neighbouring dentist in Harley Street, and told him he would bring the patient along. The anæsthetist administered the gas and the tooth was duly extracted. The question of fee then arose, and it was decided to charge an inclusive fee of half-a-crown. The patient was indignant at this, and said that her doctor in the country never charged more than a shilling. History does not relate the nature of the final settlement, but, as kindness of this sort is rarely unaccompanied by a keen sense of humour, no doubt the anæsthetist and dentist felt themselves in part repaid for their trouble in acquiring a new story at first hand for the entertainment of their friends.

### THE CARES OF THE CATERER.

THE hospital caterer is confronted by many puzzling conditions at present, and not the least of these is the difficulty in obtaining enough sugar. This particular shortage affects, by the way, also a pharmacist's department which manufactures the more simple preparations, including the various syrups. The experiences of a London hospital within the last few weeks being, no doubt, typical may be narrated. The first event was the decision of the contractor to supply only two-thirds of the average requirements. Then came an appeal to the Sugar Commission, who were supplied, before consideration of the case, with a multitude of statistics, including the sugar consumption in the year before the war, the number of patients and staff then and now, and the sources of supply. After a little delay, while the statistician of the Commission made certain calculations, arrived the welcome news that an order had been issued to the Commission's brokers to supply the hospital with 321 pounds of sugar per month until further notice, on condition that an undertaking were given that no purchases would be made outside the ordinary source of supply. Then the broker, not so precise as to weight, said that three hundredweight would be delivered upon a cash payment. A cheque was sent, and an order on a wharfinger came in exchange. The next business was to send a porter to a wharf on the Surrey side of the river to fetch the precious bags, which were brought proudly home in a taxi-cab. The sugar, it must be said, was of excellent quality and well worth the trouble; plus the cab-fare, the cost worked out at a fraction over fivepence a pound.

### THE FOOD CONTROLLER AND MILK CONTRACTS.

THE managers of the Poplar and Stepney Sick Asylum, of which Dr. Spurrell is medical superintendent, lately received a letter from the milk contractors which contained a refusal to continue to supply milk at 1s. 7d. per imperial gallon, and demanded one penny more than their previous contract price. The clerk promptly wrote to the Food Controller's Department, and received a reply that it was the duty of the managers to request the contractors to adhere to the terms of their contract, in order that they might avoid heavy penalties. Backed by the Food Controller, hospitals have little to fear from contractors who seek to revise their contracts, and his prompt support of the Poplar and Stepney institution deserves to be widely known, so that other hospitals may be encouraged to seek his advice when placed in a similar position.

### INSTITUTIONAL STAFFS AND REDUCED FOOD.

THERE have been some complaints amongst officers engaged in public institutions with regard to the reduction of their dietary in order to bring it within the scope of the scale laid down by the Food Controller. Officials in Poor-Law institutions are engaged on salary and emoluments, the latter consisting of a certain amount of food, which is valued when their superannuation allowances are taken into account at a certain amount. It is maintained by them that under the scale of the Food Controller they are liable to a financial loss, though in some instances they have overlooked the fact that the food supplied them now, though reduced in quantity, is far above the value of the scale they received in pre-war days. Lambeth Board of Guardians attempted to remedy this apparent grievance of their officers by proposing to grant them gratuities of £10 each on condition that they relinquished a previous gratuity of £4 each, but there was such a considerable amount of opposition when the Board discussed it, that it was referred back to the committee for further consideration. It was stated by the chief officers to the committee that the scale as laid down by Lord Devonport is not sufficient to sustain an able-bodied official, and that consequently they felt a loss in not being able to maintain the general standard of comfort which had hitherto obtained. It is inevitable that in these days of shortage there must be some people who feel that they cannot exist on the shortened rations, but they will have to conform to the regulations, even at the expense of some sacrifice greater than they have hitherto undergone. We cannot see that these sheltered officials have any real grievance, nor does their attitude strike us as ultra-patriotic.

### OSCAR WILDE AND HOSPITAL APPEALS.

THE association of famous men of letters with voluntary hospitals is always interesting, and it is probably not generally known that Oscar Wilde was associated with two of the early charity books, and in his journalistic days actually drafted a newspaper appeal for a hospital himself. In 1887 Mr. J. S. Wood, secretary of the Chelsea Hospital for Women in Fulham Road, edited a compilation called the "Shaksperean Show Book," which formed the official programme of the Shaksperean Show held at the Albert Hall on May 29 and the two following days. To this various well-known authors and artists contributed, including Tennyson, Browning, Wilde, Walter Crane, Cruikshank, and Val Prinsep. Wilde's contribution was a poem in four stanzas, entitled "Under the Balcony." In 1885 the Queen's Hospital for Children, Bethnal Green, then known as the North-Eastern Hospital for Children, Hackney Road, issued a collection of stories, poems, and illustrations, at the handsome charity price of 5s., entitled "In a Good Cause." On the front cover of one of the three styles in which the volume was issued was a design in black representing a woman and children knocking at a hospital gateway. Among the contributions was a poem by Wilde, with a facsimile of his signature, entitled "Le Jardin des Tuileries." But when we turn to *The Court and Society Review* for April 1887 we find a note in support of an appeal for building a new wing to the "Great Ormond Street Child's Hospital." The text of this note, which was unsigned at the time, is given in full in Mr. Stuart Mason's astonishing volume, a "Bibliography of Oscar Wilde," published by Messrs. T. Werner Laurie. The accuracy, research, and comprehensiveness of this book are extraordinary, and Mr. Stuart Mason has given a new meaning to the art of bibliography, for much of it is extremely amusing, and covers a vast amount of ground. Our only regret is that such talent and industry were not expended on a more important author than its subject, who, however, has always had the luck to be much better and much worse treated than he deserved. For the text of Wilde's appeal the curious must be referred to Mr. Mason's pages.

### A FALL IN INFANT MORTALITY.

The fact that a remarkable decrease in the infantile death-rate, which fell throughout England and Wales from 110 to 91, is revealed in a Local Government Board report issued last week should not deter the organisers of "Baby Week" in their attempt to focus public attention on the necessity of saving infant life. In spite of the reduction reported, the fact is that twelve babies die in the British Isles every hour from diseases which are largely preventable, whereas during one year of warfare the losses by death among the whole of the English-speaking forces engaged in the war was nine per hour. That it is possible to lower the infant death-rate is shown by Alderman Broadbent's experiment at Huddersfield, where, by methods which no one regarded as anything like ideal, the rate was reduced from 139 to 44 per thousand. This year's reduction is a solid tribute to the work of the infant and maternity centres, to which the reduction must largely be ascribed. There are 1,000 of these centres in the country at present, and if "National Baby Week" results in the establishment of another thousand, that will be an achievement well worth the effort. Meanwhile, it is announced that the Mayor of Croydon is offering a War Savings Certificate to each baby born in his town during "Baby Week." If he were to revise his offer so that the rewards go to the mothers whose babies are alive in the corresponding week of next year, the money would be better spent.

### AN EDITOR ON HER MERITS.

The first editor of the *Gazette* of the 4th Southern General Hospital has bidden farewell to her readers, having been responsible for the magazine for the first eleven numbers from May 1916. These magazines have a way of preserving the character which marks their beginning, and the *Plymouth Hospital Gazette* is another example of this. It seems to have found some difficulty in collecting material from the lighter side of the daily routine of the institution, but it has avoided, on the whole, the incredible silliness which a constant attempt at being funny imposes on all who are not strong enough to put the comic in its proper place. While it is true that nothing is popular except vulgarity, it is also true that our modern army is not wholly composed of illiterate persons, and the success of those gazettes which have aimed high shows the wisdom of maintaining as high a standard as is obtainable. Perhaps the new editor, inspired by what has been already accomplished, may be able to reduce the number of advertisements facing matter, as it is called, which at present somewhat spoil the magazine's appearance.

### LEGACIES FOR PLYMOUTH INSTITUTIONS.

Plymouth institutions come in for something approaching what is euphemistically termed a "windfall" under the will, just proved, of Miss Mary Eliza Plampin Elliott, of 1 Albemarle Villas, Devonport. The testatrix, among other charitable bequests, left £2,000 to Dr. Hingston's Convalescent Home, Devonport, £1,000 to the Plymouth Eye Infirmary, £1,000 to the Devonport Blind Institution, £500 to Dr. Hingston's Deaf and Dumb Institution, Devonport, and £300 to the South Devon and East Cornwall Hospital. These handsome bequests will be the more appreciated by reason of the fact that—speaking generally—West Country hospitals do not appear to be so lavishly supported by testamentary benefactions as those in the North and the Midlands.

### THIS WEEK'S DRUG MARKET.

The market has hardly recovered from the holiday feeling, and business continues very quiet with few price changes of importance. The upward tendencies of values, however, is still noticeable, although perhaps it is not quite so pronounced in face of the temporary falling-off in demand. Sarsaparilla has again become scarce, and the price has advanced. A fair business has been done in honey at high prices. The good demand for valerian root cannot be met owing to absence of supplies. Extreme prices are asked for any chamomiles available. Spanish ergot of rye is rather scarce, but Russian is available in fair quantity. The value of essence of lemon is not quite so firmly maintained. Very little business has taken place in quinine, the price of which is unchanged. The values of tartaric and citric acids are maintained, although the demand is greater. Cream of tartar is scarcer than ever and prices seem likely to advance still further. In synthetic drugs there are practically no changes of importance to record.

